# The effect of wall thickness distribution on mechanical reliability and strength in unidirectional porous ceramics

**DOI:** 10.1080/14686996.2016.1140309

**Published:** 2016-04-11

**Authors:** Jordi Seuba, Sylvain Deville, Christian Guizard, Adam J. Stevenson

**Affiliations:** ^a^ Laboratoire de Synthese et Fonctionnalisation des Ceramiques, UMR3080 CNRS/Saint-Gobain, F-84306 Cavaillon, France.; ^b^ Institut Europeen des Membranes, Universite de Montpellier 2, Place Eugene Bataillon, 34095 Montpellier Cedex 5, France.

**Keywords:** Mechanical reliability, ceramics, porous materials, Weibull, mechanical properties, 10 Engineering and structural materials\and 102Porous/Nanoporous/Nanostructured materials, 107 Glass and ceramicmaterials, 206 Conversion/transport/storage/recovery, 205Catalyst/Photocatalyst/Photosynthesis, 303 Mechanical/Physicalprocessing

## Abstract

Macroporous ceramics exhibit an intrinsic strength variability caused by the random distribution of defects in their structure. However, the precise role of microstructural features, other than pore volume, on reliability is still unknown. Here, we analyze the applicability of the Weibull analysis to unidirectional macroporous yttria-stabilized-zirconia (YSZ) prepared by ice-templating. First, we performed crush tests on samples with controlled microstructural features with the loading direction parallel to the porosity. The compressive strength data were fitted using two different fitting techniques, ordinary least squares and Bayesian Markov Chain Monte Carlo, to evaluate whether Weibull statistics are an adequate descriptor of the strength distribution. The statistical descriptors indicated that the strength data are well described by the Weibull statistical approach, for both fitting methods used. Furthermore, we assess the effect of different microstructural features (volume, size, densification of the walls, and morphology) on Weibull modulus and strength. We found that the key microstructural parameter controlling reliability is wall thickness. In contrast, pore volume is the main parameter controlling the strength. The highest Weibull modulus (m=13.2) and mean strength (198.2 MPa) were obtained for the samples with the smallest and narrowest wall thickness distribution (3.1 μm) and lower pore volume (54.5%).

## Graphical Abstract

**Figure:**
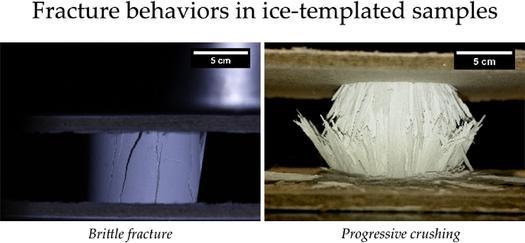

## 1. Introduction

Macroporous ceramics are used in applications such as solid oxide fuel cells (SOFC), oxygen transport membranes (OTM), bone replacement, filters, and thermal insulation [1]. In all of these cases, the functional properties must be balanced with the mechanical requirements of the application.

For brittle solids like ceramics, there is an inherent strength variability measured across seemingly identical samples, so the mean strength is not an adequate predictor of performance. Strength variability is caused by the random nature of defects created during processing, handling, or service. Since the strength of a material is described by a distribution rather than a single value, mechanical reliability must be characterized using a probabilistic approach. This is particularly important in applications like SOFC or OTM where hundreds or thousands of individual macroporous elements must be combined and the failure of a single element could cause the entire module to fail.

Although different models have been proposed to describe the strength of brittle materials [2,3], the Weibull analysis is the most extensively used [4]. It is based on the assumption that the catastrophic failure of the material is triggered by the weakest defect (i.e. *weakest link hypothesis*), and that these defects have probabilistic population densities in real materials that result in probabilistic strength distributions. One of the main implications of this assumption is that reducing the population of flaws, for example by reducing the sample size, will inevitably lead to an increase in strength [5]. Characterizing the strength distribution of a ceramic material and linking it to processing parameters and microstructural features is therefore of primary importance for industrial applications [6]. This is particularly important for macroporous ceramics because the mean strength of the materials is reduced by introducing the porosity that enables the desired functional properties.

Similar to dense brittle materials, strength in macroporous brittle ceramics can be described by linear elastic fracture mechanics [7]. This observation implies that strength is strongly dependent on the largest defect (i.e. porosity deliberately introduced) and the fracture behavior is typically catastrophic. Under these conditions, several authors [8,9] reported that the Weibull distribution describes the scattering of strength in macroporous ceramics even at high porosity (around 60%) [10], although the weakest link hypothesis assumes that the density of flaws has to be low enough to neglect the interaction between pores [11]. Nonetheless, it is worthto mention that the Weibull distribution has also been successfully applied to other types of failure that are not clearly linked to a single flaw, such as pitting corrosion in pipes [12], fatigue life of steel [4], dielectric breakdown strength [13], and adhesive wear of metals [14] or even in applications as diverse as tracking the wind speed distribution [15], recording the interoccurrence times of earthquakes [16], calculating sterility in thermal preservation methods [17], or analyzing survival data from clinical trials [18]. Thus, while the weakest-link hypothesis underpins the application of Weibull analysis to ceramic strength distributions, Weibull statistics may provide an adequate descriptor of strength distributions even in cases where failure cannot be conclusively linked to a single defect.

The reliability of macroporous ceramics has been characterized for materials processed by different techniques, e.g. partial sintering [19], organic templating [20], direct foaming [21], and robocasting [22–25]. In most cases, wall thickness has been identified as the main parameter controlling the mechanical reliability. However the particular impact of porosity on Weibull modulus is still not fully understood and frequently assumed to be the same as strength. The main problem is that most processing techniques do not provide independent control of the different features of the porous microstructure and therefore can lead to biased conclusions. For example, increasing pore volume frequently causes an increase in the pore size, hindering the assessment of the individual effect of each parameter.

Several processing methods such as wood pyrolysis, additive manufacturing, or extrusion have been developed to produce low tortuosity porous structures with controlled microstructures. These types of structure can be beneficial in applications where the tortuosity inherent to other porous ceramic processing methods is detrimental to the functional properties.

Ice-templating is a low tortuosity macroporous processing technique based on the segregation of particles caused by an advancing solidification front. After solidification is completed, the solvent is removed by sublimation and, thus, the remaining porosity is a replica of the solvent crystals. This technique has drawn the attention of different studies due to its unidirectional porosity and flexibility controlling pore volume, size, and shape [26]. The strength of these materials has been extensively studied [27–30]; however, there is a lack of studies measuring their reliability. The only work that evaluates reliability of ice-templated structures was performed by Ojuva et al. [31] in zeolites. They measured the effect of solids loading and cooling rate on Weibull modulus and estimated the probability of survival. However, the reduced number of samples tested (three to 11 samples) per condition hinders a clear interpretation of the results and a further link between microstructure and reliability.

The purpose of this work is to determine the main microstructural parameters that control the reliability and strength of unidirectional porous materials. Weibull analysis is applied to the compressive strength data gathered for different ice-templated microstructures, and we discuss the suitability of applying this model to unidirectional porous ceramics.

## 2. Experimental procedure

### 2.1. Sample preparation

Suspensions were prepared by mixing distilled water with 3 mol% yttria-stabilized zirconia (TZ-3YS, Tosoh, Tokyo, Japan) at different weight ratios (50% and 65%), 0.75 wt% of dispersant (Prox B03, Synthron, Levallois-Paris, France), and 3 wt% of organic binder PVA (PVA2810, Wacker, Burghausen, Germany). In some suspensions, zirconium acetate (20 g l${}^{-1}$) was added to the slurry to modify the pore morphology. The percentages of dispersant and binder are referred to the weight of the initial powder mass. Afterwards, the slurry was magnetically stirred to ensure a good dispersion and ball milled for a minimum of 18 h to break up the agglomerates. Then, it was deaired for at least 10 min.

The ice templating process consisted of pouring 10 ml of slurry into a PTFE mold (20 mm diameter 25 mm height) placed on a copper plate and freezing it from the bottom to the top. The top of the samples was exposed to air and kept at room temperature. The freezing temperature was controlled by circulating silicone oil regulated by a cryothermostat (Model CC 905, Hubert, Offenburg, Germany). The cooling rate was set at 2${}^{\circ }$C min${}^{-1}$. A faster cooling rate was achieved dipping a copper rod with the mold on top in liquid nitrogen. The cooling rate was monitored with a thermocouple and determined to be 25${}^{\circ }$C min${}^{-1}$ on average. After solidification, samples were removed from their molds and sublimated for at least 48 h in a commercial freeze-dryer (Free Zone 2.5 Plus, Labconco, Kansas City, MO, USA).

**Figure 1.**
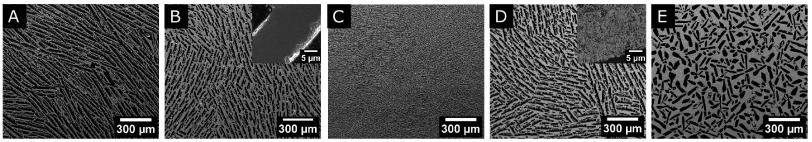
SEM micrographs obtained under the conditions specified in Table 1. (A) *S1*, (B) *S2*, (C), *S3*, (D) *S4*, and (E) *S5*.

Binder was removed from the green bodies by heating to 500${}^{\circ }$C at 3${}^{\circ }$C min${}^{-1}$ with a 5 h hold. Then, samples were sintered either at 1300${}^{\circ }$C or 1400${}^{\circ }$C at 5${}^{\circ }$C min${}^{-1}$ and held for 3 h. The cooling rate was kept constant at 5${}^{\circ }$C min${}^{-1}$ until room temperature.

### 2.2. Morphological characterization

The overall porosity P(%) of the specimens was calculated based on the mass (*m*) and volume (*V*) of the samples with respect to that of fully dense TZ-3YS ($\rho_{ysz}=5.8$ g cm${}^{-3}$), as:

(1) $$\begin{array}{cc} \rho_{rel} & =\frac{\rho }{\rho_{ysz}}=\frac{mV^{-1}}{\rho_{ysz}} \end{array}$$

(2) $$\begin{array}{cc} P(\%) & =(1-\rho_{rel})\times 100\% \end{array}$$

**Table 1.** Summary of the most relevant structural features of images in Figure 1. $d_{p}$ represents the pore size and WT the wall thickness, both obtained by image analysis. *N* is the number of tested samples.

							Freezing rate			
Label	*N*	Solids loading (wt%)	$P_{total}\left(\%\right)$	$P_{inter}\left(\%\right)$	$P_{intra}\left(\%\right)$	Morphology	(${}^{\circ }$C min${}^{-1}$)	Sintering temperature (${}^{\circ }$C)	Mean $d_{p}$ (μm)	Mean WT (μm)
S1	21	50	71.7 ± 0.4	59.7 ± 0.4	12.0 ± 0.6	Lamellar	2	1400	20.0 ± 8.5	11.2 ± 4.5
S2	23	65	53.7 ± 1.6	40.8 ± 2.1	12.9 ± 2.6	Lamellar	2	1400	13.7 ± 4.8	19.1 ± 8.2
S3	15	65	54.5 ± 1.2	40.0 ± 0.8	14.5 ± 1.4	Lamellar	25	1400	3.1 ± 1.2	3.0 ± 1.3
S4	15	60	70.5 ± 0.4	46.4 ± 0.6	24.1 ± 0.7	Lamellar	2	1300	15.0 ± 5.6	17.1 ± 8.1
S5	20	65	53.1 ± 0.7	39.1 ± 1.7	14.0 ± 1.8	Honeycomb	2	1400	27.3 ± 9.8	37.2 ± 16.7

The results were confirmed in some specimens by the Archimedes method (ASTM B962-13). The determination of interlamellar porosity $P_{inter}\left(\%\right)$, intralamellar porosity $P_{intra}\left(\%\right)$, pore size $d_{p}$, and wall thickness *WT* were performed by image analysis using the “Local thickness” plug-in of the Fiji software [32]. All the images were taken at different locations in a cross section perpendicular to the freezing direction (7 mm from the bottom of the sample) with a scanning electron microscope (Nova NanoSEM 230, FEI, Hillsboro, USA) at 10–15 kV.

### 2.3. Mechanical characterization

The mechanical properties of ice-templated samples were measured by a compression test (LR15K Plus, Lloyd Instruments, Meerbusch, Germany) with porosity aligned parallel to the load. The crush test were carried out at a crosshead speed of 0.5 mm min${}^{-1}$. Before testing, the bottom and the top of the samples were removed with a slow speed saw leaving the final dimensions around 12 mm diameter and 15 mm height. Samples were tested with a cardboard pad on both sides to minimize the effect of superficial defects and misalignment. In all the tests, the maximum load at the end of the elastic stage was used to calculate the compressive strength ($\sigma_{f}$). Afterwards, a two-parameter Weibull analysis was applied to the compressive strength data to predict the probability of failure (*$P_{f}$*) for a given stress (*σ*) through the expression:

(3) $$\begin{array}{c} P_{f}=1-exp\left[-{\left(\frac{\sigma }{\sigma_{0}}\right)}^{m}\right] \end{array}$$

where *m* is the Weibull modulus and *$\sigma_{0}$* the characteristic strength where $P_{f}$ = 0.632. To obtain an unbiased measurement of *m*, a minimum of 15 samples were tested.

**Figure 2.**
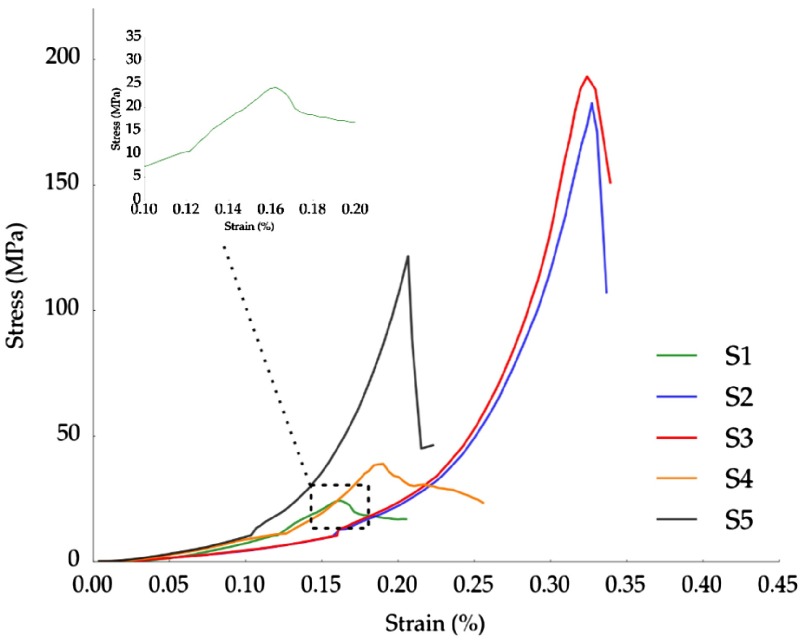
Typical stress–strain curves for samples listed in Table 1. Inset: Detail of the stress–strain curve of sample *S1*.

If we want to compare the Weibull modulus of the various samples and interpret differences between them, it is important to understand the statistical confidence interval that results from our data and fitting procedures. Therefore, we fit the data using two different techniques. First, *m* and *$\sigma_{0}$* were determined from an ordinary least squares (OLS) fit of ($LnLn(1/\left(1-P_{f}\right))$) versus ($Ln\sigma_{f}$) where the slope of the resulting line is *m* and (*$\sigma_{0}$*) is the solution to $LnLn(1/\left(1-P_{f}\right))=0$. Second, a Bayesian Markov Chain Monte Carlo (MCMC) was applied to directly fit the data to the nonlinear Equation (3).

## 3. Results

Figure 1 shows representative cross sections of the ice-templated samples for each group studied. Table 1 summarizes the most important structural features and the experimental conditions used. Figure 1b (*S2* in Table 1) was used as a reference material to evaluate the effect of different microstructural parameters on reliability.

### 3.1. Microstructural control

Total pore volume P(%) was adjusted by the solids loading. For example, decreasing the solids loading from 65 to 50 wt% caused an increase of the total pore volume from 53.7% to 71.7% (Table 1). Pore size was mainly controlled by the freezing rate. When samples were ice-templated faster (25${}^{\circ }$C min${}^{-1}$), pore size became smaller (3.1 μm), as shown in Figure 1C and Table 1. Pore morphology was modified by the addition of zirconium acetate to the initial slurry turning the lamellar pores to honeycomb-like structures (Figure 1E) [33]. Finally, we produced samples with different amounts of intralamellar porosity (i.e. porosity in the walls, $P_{intra}\left(\%\right)$). The solids loading and sintering temperature were adjusted to obtain two different groups (*S1* and *S4*) with the same total pore volume ($P_{total}\left(\%\right)$) but different densification within the walls. *S4* (Figure 1d) was sintered at lower temperature and, obviously, exhibits a higher $P_{intra}\left(\%\right)$ (12.0 ± 0.6 % compared with 24.1 ± 0.7).

### 3.2. Mechanical behavior

**Figure 3.**
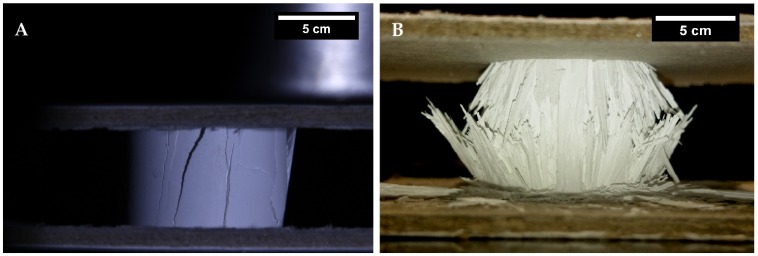
Close-up of the two different fracture behaviors observed in ice-templated samples. (A) brittle fracture sample from group *S3* and (B) progressive crushing from group *S1*.

Stress–strain curves representative of each sample set (*S1*–*S5*) are shown in Figure 2. Groups *S2*, *S3*, and *S5* exhibited a linear increase in stress up to a sudden drop, indicative of the overall fracture of the specimen. The abrupt decrease in stress can be correlated with the propagation of macrocracks parallel to the maximum loading direction that caused catastrophic failure (see Figure 3A). In contrast, groups *S1* and *S4* exhibited a different fracture behavior. After the stress reaches the peak value (i.e. strength) it drops slightly followed by a steady stage (inset in Figure 2). In these cases, samples exhibited a radial fracture at the midpoint, where the buckling stress reaches the maximum point (Figure 3B). Afterwards, samples fail by progressive crushing of the walls represented in the stress–strain curve as a plateau. In all cases, the strengths used in the statistical analysis and interpretation of the data were the maximum stresses recorded before the initial fracture.

**Figure 4.**
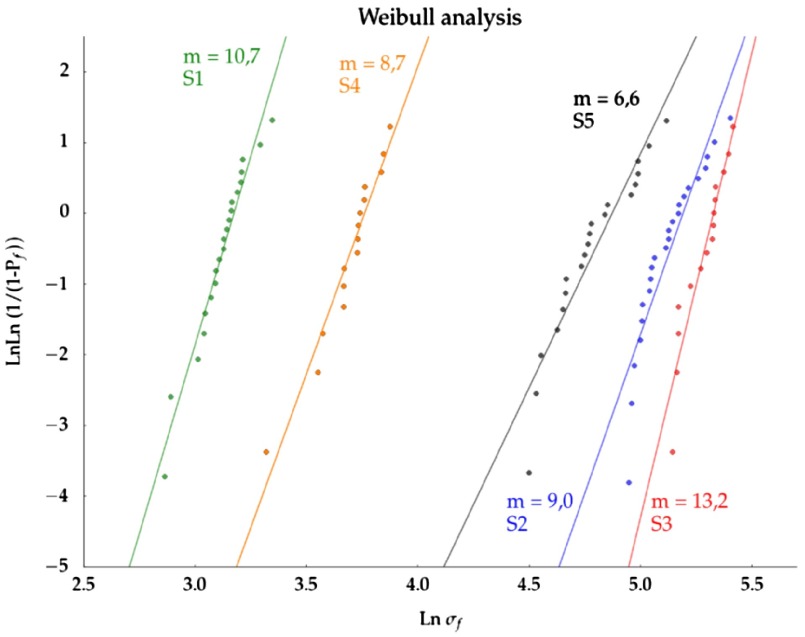
Weibull strength distributions of groups described in Table 2. Solid lines represent the OLS fit to the data.

Figure 4 shows all the experimental strength values obtained for each group of samples and the ordinary squared fit (OLS) to the data. The Weibull modulus for the compressive test of each group was 10.7 ± 0.5 (*S1*), 9.0 ± 0.8 (*S2*), 13.2 ± 1.2 (*S3*), 8.7 ± 0.6 (*S4*), and 6.6 ± 0.5 (*S5*). Additionally, Table 2 reports the mean wall thickness (WT) of the samples along with the results of the two different curve fitting procedures applied, ordinary least squares (OLS) and Bayesian fit (MCMC).

The values of $R_{ols}^{2}$ obtained for all the groups are higher than 0.85. *P*-values of the OLS fits were 0.00 and are not reported in Table 2. The combination of $P_{ols}$ = 0.00 and $R_{ols}^{2}$ > 0.85 indicates that the data in Figure 4 is well described by the linearized form of Equation (3) for all groups. Furthermore, the Weibull parameters (*m* and $\sigma_{0}$) obtained by the two different fitting methods (OLS and Bayes) are remarkably similar. Both observations indicate that the strength data presented here are adequately described by the Weibull statistical approach.

The Bayesian *p*-value was used as an indicator of the fit and it is reported in Table 2. By definition Bayesian *p*-values range between 0 and 1 and values > 0.975 or < 0.025 indicate a poor fit to the data [34]. In this case, the measured Bayesian *p*-values fall outside this range and indicate that the model and modeled *m* and *$\sigma_{0}$* parameters fit the data well and can be used in the prediction of the $P_{f}$ of this materials.

## 4. Discussion

### 4.1. Weibull modulus

Two different failure modes can be observed in Figure 2: brittle and cellular. Samples *S2*, *S3*, and *S5* clearly showed a brittle failure characterized by a sudden drop in stress. In these cases, and similarly as in dense ceramics, the Weibull analysis can be safely applied. Alternatively, *S1* and *S4* exhibited the characteristic compressive behavior of highly porous materials. As it has been reported in isotropic [35] and anisotropic [36] porous materials, the shifting behavior of the failure mode is mainly caused by the increase in pore volume. When the porosity increases, the amount of stored energy decreases and the structure becomes less prone to break by brittle failure.

**Table 2.** Summary of the results of the different curve fitting procedures (OLS and Bayes).

Label	Mean WT (μm)	$\overset{¯}{\sigma }$ (MPa)	m${}_{ols}$	m${}_{Bayes}$	$\sigma_{0\;ols}$ (MPa)	$\sigma_{0\;Bayes}$ (MPa)	$R_{ols}^{2}$	P${}_{Bayes}$
S1	11.2 ± 4.5	22.9 ± 2.5	10.7 ± 0.5	11.6 ± 0.4	23.8	23.7 ± 0.1	0.96	0.796
S2	19.1 ± 8.2	170.2 ± 22.0	9.0 ± 0.8	8.4 ± 0.5	178.9	176.9 ± 1.0	0.85	0.816
S3	3.0 ± 1.3	198.2 ± 16.9	13.2 ± 1.2	12.7 ± 0.8	202.2	206.3 ± 0.8	0.91	0.514
S4	17.1 ± 8.1	40.3 ± 5.0	8.7 ± 0.6	9.2 ± 0.5	43.4	43.0 ± 0,2	0.95	0.264
S5	37.2 ± 16.7	122.0 ± 21.5	6.6 ± 0.5	5.9 ± 0.3	133.3	129.3 ± 1.1	0.91	0.777

The presence of cellular-like failure implies that multiple fracture events occur simultaneously and thus the applicability of Weibull analysis may be questioned. However, we considered that the strength of samples in groups *S1* and *S4* is determined by a single event (i.e. when the stress reaches the buckling strength) and therefore Equation (3) can still describe the strength distribution. This behavior can be observed in the inset of Figure 2, when the stress reaches the maximum value after the initial linear step (i.e. buckling strength) and then suddenly drops. Afterwards the stress stabilizes to a constant value and the other failure events, such as progressive crushing of the struts, take place.

**Figure 5.**
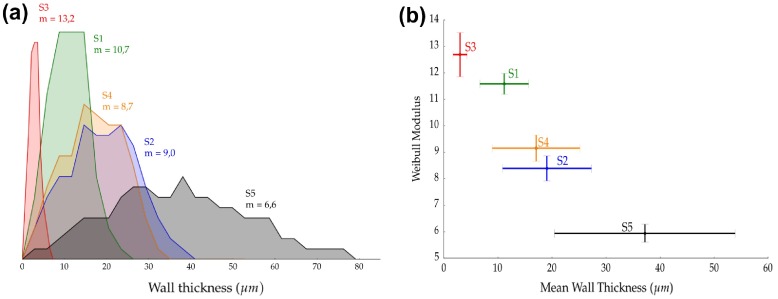
(A) Wall thickness distribution of samples shown in Figure 1 and representative of groups in Table 2. (B) Weibull modulus as a function of wall thickness. The Weibull modulus and credibility interval are taken from the Bayesian nonlinear fitting procedure.

Similar to bulk materials, Weibull modulus of macroporous ceramics is mainly determined by the size of the element where the fracture initiates. In this case, this is the ceramic struts. When the total pore volume of the specimens is equivalent, the volume of material under solicitation is also the same. However, the variation of pore size modifies the individual volume of the walls and consequently restricts the appearance of larger deleterious flaws. Accordingly, the reliability of macroporous ceramics is mainly controlled by the individual volume of the struts. Figure 5A shows that this control of wall thickness can be achieved by either solids loading (*S1*), freezing rate (*S3*), sintering temperature (*S4*), or additives in the initial slurry (*S5*). In all these instances, the mean wall thickness decreases, reducing the individual volume of the walls, and thus affecting the probability of finding a catastrophic defect and by extension the Weibull modulus. This effect can be observed in Figure 5B where Weibull modulus decreases progressively when the wall thickness increases. Additionally, decreasing mean wall thickness also narrows the wall thickness distribution. This effect decreases the standard deviation (STD) and reduces even further the probability of finding a defect. Although both mean and standard deviation certainly play an important role on the reliability, we cannot separate the individual effect of each parameter.

**Figure 6.**
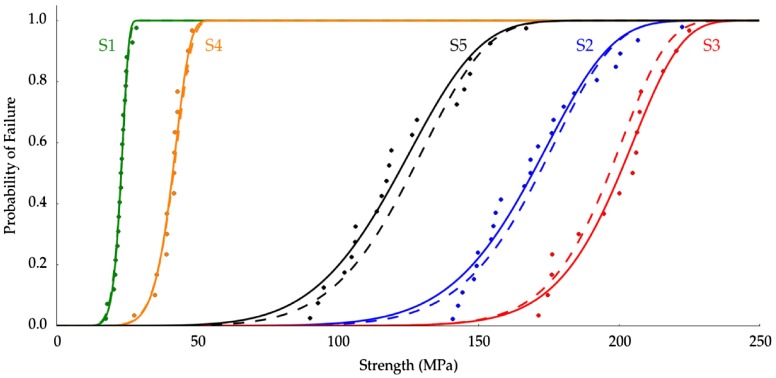
Probability of failure prediction based on the parameters $\sigma_{0}$ and *m* shown in Table 2. The dotted lines are based on the OLS fitted parameters and the solid lines use parameters from the nonlinear Bayesian fit.

Figure 6 shows the probability of failure ($P_{f}$) measured by Equation (3) and using the Weibull parameters $\sigma_{0}$ and *m* obtained through OLS and Bayesian fits (Table 2). As Figure 6 shows, when the Weibull modulus increases, the slope of the $P_{f}$ function becomes higher and the stress range between low and high probability of failure shrinks. This narrowing means that the material is more reliable, i.e. the statistical spread of sample strengths is clustered more closely around the mean value of strength distribution, and these samples can be used in operational conditions closer to the measured mean strength than a lower reliability material.

### 4.2. Mean strength

As expected, the main parameter controlling mean strength ($\overset{¯}{\sigma }$) is total pore volume ($P_{total}\left(\%\right)$). Samples *S1* and *S4* with the total pore volume of ca. 70% exhibited $\overset{¯}{\sigma }$=22.9 ± 2.5 and 40.3 ± 5.0 MPa, remarkably lower than the 170.2 ± 22.0, 198.2 ± 16.9, and 122.0 ± 21.5 MPa exhibited by samples with the total pore volume of ca. 53% (*S2*, *S3*, and *S5* respectively).

Besides total pore volume, there are also other morphological parameters that affect the strength of macroporous materials. Groups *S2*, *S3* and *S5* in Table 1 have a comparable $P_{total}\left(\%\right)$ and the mean pore size ($d_{p}$) was modified by either decreasing the freezing rate (*S3*) or changing the pore morphology (*S5*). In this case, the set of samples with the smallest pore size (*S3* in Table 2) exhibited the highest mean strength. The effect of increasing strength with decreasing pore size has been extensively reported in other ice-templated materials [31,37,38] and in other types of cellular structures [35,39–43]. Brezny and Green [44] proposed that one of the main contributions is caused by the reduction of the volume of the struts, thus affecting the probability of finding a catastrophic defect. Therefore, it becomes more appropriate to refer to a wall thickness effect rather than a pore size effect. This change in the terminology is particularly pertinent in load bearing applications where the stress distribution through the struts is of primary importance.

Finally, an increase in Weibull modulus does not necessarily lead to a higher strength. Groups *S1* and *S4* have the same total pore volume $P_{total}\left(\%\right)$, but the percentages of $P_{inter}\left(\%\right)$ and $P_{intra}\left(\%\right)$ were modified adjusting the solids loading and sintering temperature. Although *S1* has a higher reliability than *S4*, its mean strength is still lower (23.8 MPa and 43.4 MPa respectively). This effect is most likely due to the larger amount of $P_{inter}\left(\%\right)$ exhibited by *S1* that weakens the structure. Additionally, it also suggests that the strength of the individual struts is less important in the overall strength than the percentage of interlamellar porosity $P_{inter}\left(\%\right)$. Li et al. [45] found similar results experimentally and in simulations of ice-templated TiO${}_{2}$.

Although high Weibull modulus and high strength are linked in most instances, there is a fundamental difference between both. While the latter positions a value in the strength distribution (i.e. $\overset{¯}{\sigma }$ is strength with a $P_{f}=50\%$ and $\sigma_{0}$ at $P_{f}=63\%$), the former determines the spread of the distribution and sometimes is referred to even as a shape parameter. Thus, the strength and reliability are properties that might be controlled almost separately [46]. These phenomena can be clearly observed comparing groups *S2* and *S4*. Both exhibited a similar wall thickness distribution and consequently a comparable Weibull modulus (9.0 and 8.7 respectively), but their strengths were radically different due to the large differences in $P_{total}\left(\%\right)$.

## 5. Conclusions

The mechanical reliability of ice-templated specimens was measured in compression in different pore structures through a Weibull analysis. Two fundamentally different fitting methods (OLS and Bayesian) have been successfully applied and give similar values for both *m* and *$\sigma_{0}$*. Further, the diagnostic parameters ($R^{2}$ and *p*-value) for both fitting methods indicate that the data are well described by Equation (3).

We also observed that in ice-templated materials strength and reliability (*m*) are properties that can be controlled quasi-independently. Weibull modulus exhibited a strong dependency on wall thickness, which we attributed to the reduced probability of finding a catastrophic defect in thinner walls. In contrast, the strength is mainly determined by the total pore volume ($P_{total}\left(\%\right)$). Morphological parameters like wall thickness and interlamellar porosity ($P_{inter}\left(\%\right)$) also affect strength, but far more weakly than total porosity.

The possibility to aim for specific strength (through $P_{inter}\left(\%\right)$) and tailor the Weibull modulus (through the wall thickness distribution) is a powerful tool for porous materials in load bearing applications where we must combine high reliability with mechanical stability. The capacity of ice-templating to tailor the percentage of inter- and intra- lamellar porosity individually provides a microstructural control that might be useful in biomedical and energy applications where both types of porosity are required [47–49].
